# Association of affective temperaments with blood pressure and arterial stiffness in hypertensive patients: a cross-sectional study

**DOI:** 10.1186/s12872-016-0337-9

**Published:** 2016-08-08

**Authors:** Andrea László, Ádám Tabák, Beáta Kőrösi, Dániel Eörsi, Péter Torzsa, Orsolya Cseprekál, András Tislér, György Reusz, Zsófia Nemcsik-Bencze, Xénia Gonda, Zoltán Rihmer, János Nemcsik

**Affiliations:** 1 Department of Family Medicine, Semmelweis University, Budapest, Hungary; 2 1st Department of Medicine, Semmelweis University, Budapest, Hungary; 3 Department of Epidemiology and Public Health, University College, London, UK; 4 1st Department of Pediatrics, Semmelweis University, Budapest, Hungary; 5 Magnetic Resonance Imaging Research Center, Semmelweis University, Budapest, Hungary; 6 Department of Pharmacodynamics, Semmelweis University, Budapest, Hungary; 7 Department of Psychiatry and Psychotherapy, Semmelweis University, Budapest, Hungary; 8 MTA-SE Neurochemistry Research Group, Budapest, Hungary; 9 Health Service of Zugló (ZESZ), Budapest, Hungary

**Keywords:** Affective temperament scores, Blood pressure, Arterial stiffness, Augmentation index, Hypertension

## Abstract

**Background:**

Affective temperaments (anxious, depressive, cyclothymic, irritable and hyperthymic) measure subclinical manifestations of major mood disorders. Furthermore, cumulating evidence suggests their involvement in somatic disorders as well. We aimed to assess associations between affective temperament scores and blood pressure and arterial stiffness parameters in hypertensive patients.

**Methods:**

In this cross-sectional study, 173 patients with well-controlled or grade 1 chronic hypertension, with no history of depression, completed the TEMPS-A, Beck Depression Inventory (BDI) and Hamilton Anxiety Scale (HAM-A) questionnaires in three GP practices. Arterial stiffness was measured with tonometry (PulsePen).

**Results:**

According to multiple linear regression analysis, cyclothymic temperament score was positively associated with brachial systolic blood pressure independently of age, sex, total cholesterol, brachial diastolic blood pressure, BDI, HAM-A and the use of alprazolam (*β* = 0.529, *p* = 0.042), while hyperthymic temperament score was negatively related to augmentation index independent of age, sex, smoking, heart rate, BDI, HAM-A and the use of alprazolam (*β* = -0.612, *p* = 0.013). A significant interaction was found between cyclothymic temperament score and sex in predicting brachial systolic blood pressure (*p* = 0.025), between irritable and anxious temperament scores and sex in predicting pulse wave velocity (*p* = 0.021, *p* = 0.023, respectively) and an interaction with borderline significance between hyperthymic temperament score and sex in predicting augmentation index (*p* = 0.052).

**Conclusions:**

The present findings highlight elevated blood pressure among subjects with high cyclothymic temperament as well as an increased level of arterial stiffening in subjects with low hyperthymic scores suggesting that affective temperaments may play a role in the development of hypertension and arterial stiffening and may thus represent markers of cardiovascular risk. Sex differences were also present in these associations.

## Background

Cardiovascular diseases are the leading cause of morbidity and mortality in most industrialized countries worldwide, despite highly effective preventive treatments. A continuous linear relationship between elevated blood pressure and incident cardiovascular events is well known at all ages and in all ethnic groups [1–5]. In addition to elevated blood pressure, arterial stiffening – integrating the damage of risk factors on the aortic wall over a long period [6] – is increasingly recognized as a marker and mediator of cardiovascular diseases. Accordingly, carotid-femoral pulse wave velocity (PWV), an accepted non-invasive measure of arterial stiffness, is recommended for cardiovascular risk prediction among hypertensive patients in European Guidelines [7].

Depression is also a common public health problem in the Western world and its strong connection with cardiovascular diseases is broadly recognized [8]. The pathophysiologies of depression and cardiovascular diseases show several similarities that include the dysregulation of metabolic, immune-inflammatory and autonomic systems as well as the hypothalamic-pituitary axis [9]. In addition to depression, anger, hostility and anxiety – the negative impact of adverse individual psychological traits and characteristics – are also well-documented risk factors of coronary heart diseases [10, 11], while antagonism-related traits also appear to predict a variety of cardiovascular outcomes [12, 13].

Temperament is regarded as an inherited part of personality and represents the biologically stable core of emotional reactivity [14, 15]; however, there is ongoing discussion regarding the influence of age on depressive temperament with differences between man and women also being present [16]. Affective temperaments can be measured on five temperament scales by the Temperament Evaluation of Memphis, Pisa, Paris and San Diego Autoquestionnaire (TEMPS-A) [17]. Hyperthymic temperament is characterized by upbeat, overconfident and over-energetic traits while depressive temperament is self-denying, striving to live in harmony with others and sensitive to suffering. Anxious temperament can best be explained by exaggerated worries especially toward family members. Cyclothymic temperament shows affective instability with rapid mood shifts and intense emotions, while irritable temperament incorporates skeptical and critical traits [15, 18, 19]. Affective temperaments are also associated with numerous other measures of psychopathology [20]. Specific affective temperament types are the subclinical, trait-related manifestations and commonly the antecedents of minor and major mood disorders [21], where hyperthymic affective temperament can inversely be related to depression [22]. Recently, we described an association between chronic hypertension and the dominant cyclothymic temperament [23]. Cyclothymic temperament was also associated with acute coronary events in hypertensive patients [24]. In a small matched case-control study comparing hypertensive patients with and without dominant affective temperaments, we found lower peripheral and central diastolic blood pressure values and decreased serum brain-derived neurotrophic factor levels in patients with dominant temperaments [25]. Since the ratio of those subjects who score high on different temperament directions while having a dominant affective temperament is relatively low (rarely does a subject have multiple dominant temperaments), as found in only 16.8 % of the general population [26], it would be important to ascertain the continuous association between affective temperament scores and blood pressure or arterial stiffness parameters.

We hypothesized that individual affective temperament scores may be related to brachial blood pressure as well as arterial stiffness in chronic hypertensive patients. We speculated a positive association in instances of depressive, cyclothymic, irritable or anxious temperaments and an inverse association in instances of hyperthymic temperament. We also hypothesized the presence of sex differences in relation to these studied associations.

## Methods

### Patients

In the present cross-sectional study, 173 Caucasian patients with well-controlled or grade 1 chronic (on medication for >3 months) hypertension were investigated in three primary care practices. Patients with atrial fibrillation and treated depression or with dementia potentially interfering with the completion of questionnaires were excluded. Patients on moderate doses of alprazolam (<0.5 mg/day) were not excluded. All of the 48 patients of our previous pilot study [25] were also involved in the present study.

### Evaluation of affective temperaments, depression and anxiety

The *Temperament Evaluation of Memphis, Pisa, Paris and San Diego Autoquestionnaire* (TEMPS-A) was used to assess affective temperaments on depressive, cyclothymic, hyperthymic, irritable and anxious subscales, requiring ‘yes’ (score 1) or ‘no’ (score 0) answers [17]. TEMPS-A contains 110 items (109 in the version for males) and the questions of the various temperament types are grouped together as follows:

1. depressive temperament: questions 1 to 21 (21 points)
2. cyclothymic temperament: questions 22 to 42 (21 points)
3. hyperthymic temperament: questions 23 to 63 (21 points)
4. irritable temperament: questions 64 to 84 (21 points in women, 20 in the men’s version)
5. anxious temperament: questions 85 to 110 (26 points).

TEMPS-A has been extensively studied, translated into more than 25 languages and validated in several of the latter. Similarities and differences were also found in national samples which suggest that distribution of affective temperaments has both universal and cultural-specific characteristics [16].

The *Beck Depression Inventory* (BDI), created by Aaron T. Beck, is a 21-question multiple-choice self-report questionnaire and is one of the widely used instruments for measuring depression severity. Participants are asked to make ratings on a four point scale, where a higher score correlates with more severe depression [27].

The *Hamilton Anxiety Scale* (HAM-A) was used to study the severity of anxiety. The scale consists of 14 items, each item is scored on a scale of 0 (not present) to 4 (severe anxiety) [28].

### Blood pressure and arterial stiffness measurements

Measurements were performed in a temperature-controlled room, between 7.00 and 8.00 a.m. prior to blood collection. Patients were required to fast overnight and refrain from smoking and drinking caffeine-containing beverages before the procedure, but to take their usual blood pressure medication. Upon arrival and after 5 min rest, two brachial blood pressure measurements were taken on each arm in the sitting position with a validated oscillometric blood pressure device (Omron M3). The mean value of the higher side of arms was further taken into calculation as brachial systolic (SBPbrach) and diastolic (DBPbrach) blood pressures and heart rate. Brachial pulse pressure (PPbrach) was also calculated from these data. The subjects were next fitted with arterial stiffness measurement device and were asked to rest in the supine position for approximately 15 min before being measured. Arterial stiffness parameters were evaluated with the gold-standard tonometric method (PulsePen, DiaTecne, Milan, Italy) [29]. This method provides estimates of pulse wave velocity (PWV) and in which central systolic blood pressure (SBPcentr), central pulse pressure (PPcentr) and pulse pressure amplification (PPAmp) can be calculated. Augmentation index (Aix), a widely used wave reflection parameter, can also be measured by automatic identification of the “first shoulder” (inflection point) of the averaged carotid pulse signal by the PulsePen software. This index is provided by the pressure amplitude following this point divided by the pulse pressure and calculated as a percentage. In these calculations, brachial blood pressure values measured in the supine position were used, which were required for calibration after each (carotid or femoral) pulse wave detection. In each subject, two sequences of arterial stiffness measurements were performed and their mean used for statistical analysis. In the PWV calculations, 80 % of the carotid-femoral distance was used, according the most recent recommendation [30]. The intra- and interobserver variability of PWV measurements obtained by the PulsePen device in hypertensive patients was 4.6 and 6.3 %, respectively. Since PulsePen calculates pressures based on brachial diastolic blood pressure calibration, the calculated central diastolic blood pressure is identical to the brachial diastolic blood pressure assessed in the supine position [29].

### Statistical analysis

Descriptive data are expressed as mean ± standard deviation or median with interquartile ranges or percentages. Normality of continuous parameters was tested with the Kolmogorov-Smirnov test. Pearson correlation coefficients were calculated to study the relationship between affective temperament scores and demographic, hemodynamic or arterial stiffness parameters. Multiple linear regression analysis was used to study the determinants of these hemodynamic or arterial stiffness parameters which were associated in univariate analysis with affective temperaments. Based on literature data, sex differences in the association between affective temperaments and the studied hemodynamic or arterial stiffness parameters [16] were expected, and therefore sex and its interaction with the given affective parameter was included into all regression models and where an interaction was found, such interaction was further studied. A two-sided *p* < 0.05 was considered to be significant. SPSS 13.0 for Windows was used for all calculations.

## Results

A total of 173 subjects were included. Baseline demographic and laboratory parameters, current medication, TEMPS-A, BDI and HAM-A scores, central blood pressure and arterial stiffness parameters are summarized in Table 1. The median number of antihypertensive drugs taken was 2 (IQR: 2-3).

**Table 1:** Baseline characteristics of study participants

N (male/female)	173 (68/105)
Age (years)	63 (53-70)
Duration of hypertension (year)	9 (3-16)
Diabetes [*n* (%)]	38 (22)
CV disease [*n* (%)]	26 (15)
Current smoker [*n* (%)]	33 (19.1)
Body height [cm]	168 (160-174)
Body weight [kg]	80 (70-90)
BMI [kg/m^2^]	27.8 (25.3-31.2)
Blood glucose [mmol/l]	5.6 (5.1-6.6)
GFR-EPI [ml/min/1.73 m^2^]	81.9 (67.9-90)
Uric acid [μmol/l]	324.2 ± 79.6
Total cholesterol [mmol/l]	5.2 ± 1.1
Triglyceride [mmol/l]	1.4 (1.1-2.1)
Medications	
ACE-inhibitor [*n* (%)]	116 (67.1)
ARB [*n* (%)]	35 (20.2)
Calcium channel blocker [*n* (%)]	88 (50.9)
Beta-blocker [*n* (%)]	98 (56.6)
Diuretic [*n* (%)]	72 (41.6)
Antiplatelet drug [*n* (%)]	52 (30.1)
Statin [*n* (%)]	57 (33.4)
Alprazolam [*n* (%)]	23 (13.3)
TEMPS-A, BDI, HAM-A scores	
Depressive	6 (4-9)
Cyclothymic	3 (1-5)
Hyperthymic	12 (9-14)
Irritable	3 (2-6)
Anxious	4 (2-9)
BDI	5 (2-9)
HAM-A	5 (2-10)
Hemodynamic. arterial stiffness parameters	
Heart rate [1/min]	70.8 (64.8-78)
SBPbrach [mmHg]	133.5 ± 12
DBPbrach [mmHg]	75.6 ± 9.2
PPbrach [mmHg]	54.2 (47.1-62.4)
SBPcentr [mmHg]	123 (113.2-130.8)
PPcentr [mmHg]	51 (43.5-60.4)
PPAmp	1.07 (1.00-1.13)
PWV (m/sec)	8.7 (7.7-9.9)
Aix (%)	15.5 (8.5-25.2)

Data are presented as mean ± SD or median (interquartile range). Categorical parameters are presented as n (%). *CV diseases* cardiovascular diseases, *BMI* body mass index, *GFR-EPI* glomerular filtration rate assessed by the chronic kidney disease epidemiology collaboration glomerular filtration rate equation, *ACE* Angiotensin converting enzyme, *ARB* angiotensin II receptor blocker, *TEMPS-A* Temperament Evaluation of Memphis Pisa, Paris and San Diego questionnaire, *BDI* Beck Depression Inventory, *HAM-A* Hamilton Anxiety Scale, *SBPbrach* brachial systolic pressure, *DBPbrach* brachial diastolic pressure, *PPbrach* brachial pulse pressure, *SBPcentr* central systolic pressure, *PPcentr* central pulse pressure, *PPAmp* pulse pressure amplification, *PWV* carotid-femoral pulse wave velocity, *Aix* augmentation index

Table 2 lists the hemodynamic or arterial stiffness parameters and their significant correlations for which affective temperaments were also significantly associated. Table 2 also shows those variables which were not significantly correlated with outcome variables, but were entered into the final multiple regression models. Partial correlations corrected for age and sex are also demonstrated. Although, in univariate models, affective temperament scores were associated with hemodynamic or arterial stiffness parameters in many cases, however, upon further correction for age and sex, certain temperaments failed to be independent covariables of these parameters, notably irritable temperament score of brachial systolic blood pressure (*p* = 0.056) and depressive temperament score of Aix (*p* = 0.595). Table 3 demonstrates that cyclothymic temperament score was an independent covariate of brachial systolic blood pressure and hyperthymic temperament of Aix after adjustment for further relevant confounders. In the final model adjusted for all potential confounders, a one-unit increase in cyclothymic score was associated with 0.529 (95 % CI: 0.019-1.040) mmHg higher brachial systolic blood pressure while a one-unit increase in hyperthymic score was associated with -0.612 (95 % CI: -1.092--0.132) % lower Aix.

**Table 2:** Variables with significant Pearson correlations and variables entered in the final multiple linear regression model showing the independent predictors of brachial systolic blood pressure, pulse wave velocity and augmentation index

Variable	R	*p*	Partial R^a^	*p*
Brachial systolic blood pressure				
Sex	-0.155	0.048	-	-
Cholesterol [mmol/l]	-0.179	0.022	-0.072	0.365
DBPbrach [mmHg]	0.428	<0.001	0.474	<0.001
PPbrach [mmHg]	0.484	<0.001	0.489	<0.001
SBPcentr [mmHg]	0.591	<0.001	0.598	<0.001
DBPcetr [mmHg]	0.284	<0.001	0.329	<0.001
PPcentr [mmHg]	0.461	<0.001	0.471	<0.001
SBP amplification [mmHg]	0.281	<0.001	0.300	<0.001
PWV [m/s]	0.261	<0.001	0.265	0.001
TEMPS-A Irritable	0.171	0.030	0.151	0.056
TEMPS-A Cyclothymic	0.167	0.032	0.171	0.030
Age	0.037	0.630	-	-
BDI	0.006	0.932	0.026	0.738
HAM-A	0.062	0.430	0.082	0.299
Alprazolam	0.007	0.932	0.018	0.820
Pulse wave velocity				
Age [year]	0.544	<0.001	-	-
Duration of hypertension [year]	0.250	<0.001	-0.019	0.808
CV disease	0.241	0.001	0.095	0.218
Blood glucose [mmol/l]	0.213	0.005	0.128	0.097
GFR-EPI [ml/min/1.73 m^2^]	- 0.308	<0.001	-0.112	0.154
SBPbrach [mmHg]	0.260	<0.001	0.265	0.001
PPbrach [mmHg]	0.507	<0.001	0.408	<0.001
SBPcentr [mmHg]	0.410	<0.001	0.369	<0.001
PPcentr [mmHg]	0.478	<0.001	0.338	<0.001
TEMPS-A Irritable	0.156	0.040	0.173	0.025
TEMPS-A Anxious	0.157	0.039	0.156	0.043
BDI	0.164	0.031	0.104	0.176
HAM-A	0.173	0.024	0.179	0.021
Sex	-0.143	0.059	-	-
Alprazolam	0.121	0.111	0.078	0.308
Augmentation index				
Age [year]	0.203	<0.001	-	-
Sex	0.347	<0.001	-	-
Current smoking [p/y]	0.159	0.038	0.175	0.023
Body height [cm]	0.247	0.001	-0.021	0.791
Heart rate [1/min]	-0.195	0.013	-0.151	0.058
Uric acid [μmol/l]	-0.255	<0.001	-0.163	0.035
TEMPS-A Depressive	0.168	0.027	0.041	0.595
TEMPS-A Hyperthymic	-0.215	0.004	-0.158	0.034
BDI	0.054	0.478	-0.097	0.210
HAM-A	0.040	0.605	-0.057	0.467
Alprazolam	0.048	0.525	-0.023	0.768

^a^Partial R: partial correlation coefficient, corrected for age and sex. See Table 1 for the rest of abbreviations

**Table 3:** Predictive value of cyclothymic affective temperament scores on brachial systolic blood pressure and of hyperthymic affective temperament scores on augmentation index in the various models. The progressive involvement of variables into models and other significant predictors in the final models are also demonstrated

Model	B	Std. Error	Std. Beta	*P*	Adj. R^2^
Brachial systolic blood pressure					
Model 1					0.023
Cyclothymic temp. score	0.539	0.247	0.171	0.030	
Model 2: Model 1 + Age + Sex					0.041
Cyclothymic temp. score	0.568	0.245	0.180	0.022	
Model 3: Model 2 + DBPbrach					0.245
Cyclothymic temp. score	0.464	0.218	0.147	0.034	
Model 4: Model 3 + Triglyceride + Cholesterol					0.275
Cyclothymic temp. score	0.431	0.214	0.137	0.045	
Model 5: Model 4 + BDI + HAM-A + Alp					0.269
Cyclothymic temp. score	0.529	0.258	0.167	0.042	
Age	0.177	0.076	0.181	0.021	
DBPbrach	0.629	0.094	0.482	<0.001	
Triglyceride	-1.981	0.987	-0.141	0.047	
Augmentation index					
Model 1					0.045
Hyperthymic temp. score	-0.733	0.255	-0.227	0.004	
Model 2: Model 1 + Age + Sex					0.187
Hyperthymic temp. score	-0.509	0.239	-0.158	0.034	
Model 3: Model 2 + Smoking					0.209
Hyperthymic temp. score	-0.562	0.237	-0.174	0.019	
Model 4: Model 3 + Heart rate					0.231
Hyperthymic temp. score	-0.555	0.234	-0.172	0.019	
Model 5: Model 4 + Uric acid					0.243
Hyperthymic temp. score	-0.523	0.232	-0.162	0.026	
Model 6: Model 5 + BDI + HAM-A + Alp					0.244
Hyperthymic temp. score	-0.612	0.243	-0.189	0.013	
Age	0.297	0.087	0.258	0.001	
Sex	7.445	2.189	0.264	0.001	
Smoking	6.159	2.610	0.176	0.020	
Heart rate	-0.209	0.098	-0.152	0.035	

*Std. Error* standard error, *Std. Beta* Standardized Beta, *Adj. R* ^*2*^ adjusted R^2^, *Cyclothymic temp. score* cyclothymic affective temperament score, *DBPbrach* brachial diastolic blood pressure, *BDI* Beck Depression Inventory, *HAM-A* Hamilton Anxiety Scale, *Alp* patients regularly using alprazolam, *Hyperthymic temp. score* Hyperthymic affective temperament score

With regard to the association between PWV and irritable temperament score, the correlation still remained significant (*p* = 0.012) after adjustment for age, sex, brachial systolic blood pressure, GFR-EPI, blood glucose and duration of hypertension, although became non-significant after further adjustment for BDI and HAM-A scores and the use of alprazolam (*p* = 0.078). The same results were also found for anxious temperament score and PWV: the significant association (*p* = 0.043) that was present after adjustment for age, sex, brachial systolic blood pressure, GFR-EPI, blood glucose and duration of hypertension disappeared after further adjustment for BDI and HAM-A scores and the use of alprazolam (*p* = 0.475).

**Fig. 1:**
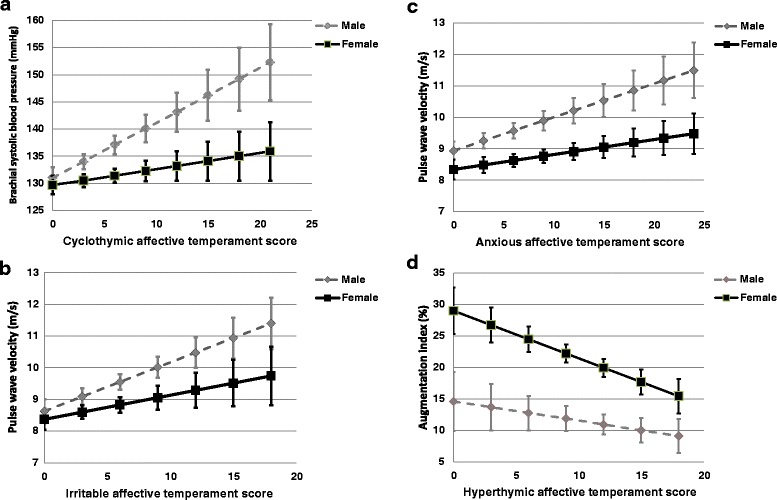
The interaction between sex and different affective temperaments in the prediction of the studied variables. **a** cyclothymic temperament score and brachial systolic blood pressure; **b** irritable temperament score and pulse wave velocity; **c** anxious temperament score and pulse wave velocity; **d** hyperthymic temperament score and augmentation index. Error bars represent ± 1 standard errors

When studying the interaction between cyclothymic temperament score and sex in predicting brachial systolic blood pressure, a significant association was found (*p* = 0.025, Fig. 1a). There was a positive association between cyclothymic temperament score and brachial systolic blood pressure in men (*B* = 1.012, *SE* = 0.392, *p* = 0.011) which was absent in women (*B* = 0.294, *SE* = 0.311, *p* = 0.346). After adjustment for age, brachial diastolic blood pressure, cholesterol and triglycerides, this interaction became non-significant (*p* = 0.090; in men *B* = 0.680, *SE* = 0.347, *p* = 0.052 and in women *B* = 0.279, *SE* = 0.272, *p* = 0.307).

There was also a significant interaction between irritable temperament score and sex in predicting PWV (*p* = 0.021). There was a positive association between irritable temperament score and PWV in men (*B* = 0.154, *SE* = 0.060, *p* = 0.012) which was absent in women (*B* = 0.076, *SE* = 0.064, *p* = 0.235) (Fig. 1b). After adjustment for age, blood glucose, brachial systolic blood pressure and GFR-EPI, the interaction p-value was attenuated (*p* = 0.037), however the strength of the association remained similar (in men *B* = 0.104, *SE* = 0.050, *p* = 0.039 and in women *B* = 0.082, *SE* = 0.052, *p* = 0.116). The interaction became non-significant (*p* = 0.168) after further adjustment for BDI and HAMA-A scores and the regular use of alprazolam (in men *B* = 0.091, *SE* = 0.051, *p* = 0.078 and in women *B* = 0.054, *SE* = 0.061, *p* = 0.375).

Similarly to irritable temperament, there was also a significant interaction between the anxious temperament score and sex in predicting PWV (*p* = 0.023). There was a positive association between anxious temperament score and PWV in men (*B* = 0.106, *SE* = 0.043, *p* = 0.015) which was absent in women (*B* = 0.047, *SE* = 0.036, *p* = 0.189) (Fig. 1c). After adjustment for age, blood glucose, brachial systolic blood pressure and GFR-EPI, the interaction p-value was attenuated (*p* = 0.046), however the strength of the association remained similar in men (in men *B* = 0.088, *SE* = 0.036, *p* = 0.017 and in women *B* = 0.021, *SE* = 0.030, *p* = 0.484). The interaction became non-significant (*p* = 0.135) after further adjustment for BDI and HAMA-A scores and the regular use of alprazolam (in men *B* = 0.070, *SE* = 0.039, *p* = 0.075 and in women *B* = -0.017, *SE* = 0.037, *p* = 0.656).

An interaction with borderline significance (*p* = 0.052) was found between sex and hyperthymic affective temperament in predicting Aix. An inverse association was found in women (*B* = -0.754, *SE* = 0.326, *p* = 0.022) which was absent in men (*B* = -0.305, *SE* = 0.370, *p* = 0.411) (Fig. 1d). This interaction became weaker (*p* = 0.064) after further adjustment for age, smoking, heart rate and uric acid (in women *B* = -0.678, *SE* = 0.312, *p* = 0.032 and in men *B* = -0.327, *SE* = 0.352, *p* = 0.325).

## Discussion

To the best of our knowledge, this is the first study to demonstrate that, in chronic hypertensive patients, cyclothymic temperament score is associated with brachial systolic blood pressure while hyperthymic temperament score is independently related to the augmentation index after adjustment for potential confounders including severity of depression and anxiety and the use of alprazolam. Sex differences were also found in relation with brachial systolic blood pressure and cyclothymic temperament score, pulse wave velocity and irritable and anxious temperament scores and augmentation index and hyperthymic temperament score.

Previous findings support our present observations that affective temperaments are associated with cardiovascular pathology. For example, patients with cyclothymic, irritable and anxious temperaments were shown to have a tendency to obesity [31]. Depressive temperament was also found to be associated with worse metabolic control in type-2 diabetes [32], while anxious temperament was associated with an increased likelihood for the presence of prediabetic condition [33]. Our result with regard to cyclothymic temperament is in line with our previous findings in which dominant cyclothymic temperament demonstrated a significant association with the presence of hypertension [23] and with acute coronary events in a hypertensive patient population [24].

An increasing cyclothymic temperament score was found herein to be associated with a higher brachial systolic blood pressure. This temperament shows a central dimension that includes rapid fluctuations in mood and emotional instability. Such alterations can span from lethargy to eutonia, from pessimistic brooding to optimistic, from hypersomnia to decreased need for sleep or from introverted self-absorption to uninhibited people-seeking [18]. In contrast, hyperthymic temperament, which was associated in our study with better wave reflection, can be described as a temperament that displays extroversion, emotional intensity, a high level of life-energy and little need for sleep. Subjects with hyperthymic temperament are cheerful, overoptimistic, overconfident as well as over-talkative and vigorous [18]. While the hyperthymic temperament is associated with better quality of life (QOL), the cyclothymic temperament is conversely associated with worse QOL [18]. Moreover, people with hyperthymic temperament can better cope with somatic problems [34], while cyclothymic disposition is related to a high somatic risk [35]. Based on these results, we hypothesize that there is a likely differential impact of these two temperaments on vascular pathology, although prospective studies are required to confirm this hypothesis.

There are existing data in the literature regarding the association between personality traits and arterial stiffening. In a study by Midei and Matthews, higher trait anxiety and hostility were associated with a higher PWV [36]. In the Baltimore Longitudinal study of Aging, middle-aged adults with suppressed anger had elevated carotid arterial stiffness [37]. In keeping with these studies, irritable and anxious affective temperament scores were found herein to be covariates of PWV after adjustment for age, sex, brachial systolic blood pressure, blood glucose, GFR-EPI and duration of hypertension. However, these associations became non-significant after further adjustment with severity of depression and anxiety and the use of alprazolam. Given that arterial stiffness is associated with depression and anxiety [38, 39], we can hypothesize that, similarly to the relationship between life stress and arterial stiffness [40], the association between anxious and irritable affective temperaments and PWV is partly mediated by severity of depressive and anxiety symptoms.

Augmentation index is an accepted parameter of pulse wave analysis. Aix is associated with age, sex, body height, smoking and heart rate [41, 42], associations which were also reproduced in the present study. Aix is furthermore reported to be a predictor of mortality in various pathological conditions, such as end-stage renal disease [43] or coronary artery disease [44], and its predictive value was also confirmed by a meta-analysis [45]. In the study of Seldenrijk et al., anxiety-related symptoms were found to be associated with Aix [39]. Our present results indicate that hyperthymic affective temperament score is an independent covariable of Aix with higher scores being associated with better wave reflection and thus a preserved elasticity of the arteries. This suggests a protective role of hyperthymic temperament on cardiovascular pathology and emphasizes the potential of further evaluation of affective temperaments with wave reflection parameters.

Significant interactions were also found in the present study between sex and cyclothymic temperament score in predicting brachial systolic blood pressure, between sex and irritable temperament as well as between sex and anxious temperament scores in predicting PWV, while the interaction between sex and hyperthymic temperament score in predicting Aix had borderline significance. These findings are consistent with the study of Williams et al., where trait anger was associated with elevated arterial stiffness in men, while in women the association was marginally significant [46]. These results suggest that, similarly to the presence of sex differences in scoring in different affective temperament directions [16], sex differences are also present in the associations between affective temperament scores and brachial systolic blood pressure and arterial stiffness parameters. Additional studies are likely needed to specify these observed sex differences, including taking into consideration menopausal status or the use of hormone replacement therapy.

The pathophysiological background of our findings is complex and remains to be clarified. Subjects in states of anger or hostility show elevated levels of inflammation [47, 48] and those with anxiety show reduced autonomic nervous system function [49]. The involvement of neurotrophic molecules should also be considered since we recently demonstrated decreased serum brain-derived neurotrophic factor levels in chronic hypertensive patients with dominant cyclothymic, depressive, anxious or irritable affective temperament [25]. However, this area also requires further studies.

One of the limitations of the present analysis is the relatively low number of chronic hypertensive patients used to study the associations between affective temperaments and blood pressure or arterial stiffness parameters, which limits the generalizability of our results, as well as the number of entered confounders into regression analyses. Another main limitation stems from its cross-sectional design which precludes causal inference. Moreover, although our methodology used standardized questionnaires and excluded patients with dementia, a complete exclusion of misinterpretations or mistakes by patients is nevertheless impossible.

## Conclusions

In conclusion, the elevated blood pressure among subjects with high cyclothymic temperament and the increased level of arterial stiffening in subjects with low hyperthymic scores suggest that affective temperaments might play a role in the development of hypertension and arterial stiffening and thus could represent potential markers of cardiovascular risk. The discovered sex differences in numerous studied associations may be the consequence of the known differences in affective temperaments between men and women. Cumulating data suggest that the identification of affective temperaments in the future can improve both the psychopathological and cardiovascular risk stratification of patients leading to a more accurate, personalized patient management.

## Abbreviations

AIx, augmentation index; ARB, angiotensin II receptor blocker; BDI, Beck depression inventory; BMI, body mass index; Cyclothymic temp. score, cyclothymic affective temperament score; DBPbrach, brachial diastolic blood pressure; GFR-EPI, glomerular filtration rate assessed by the chronic kidney disease epidemiology collaboration glomerular filtration rate equation; HAM-A, Hamilton anxiety scale; HR, heart rate; Hyperthymic temp. score, hyperthymic affective temperament score; PPAmp, pulse pressure amplification; PPB, brachial pulse pressure; PPbrach, brachial pulse pressure; PPcentr, central pulse pressure; PWV, pulse wave velocity; SBPbrach, brachial systolic blood pressure; SBPcentr, central systolic blood pressure; TEMPS-A, the temperament evaluation of Memphis Pisa, Paris and San Diego questionnaire
